# In vitro fertilization and long-term child health and development: nationwide birth cohort study in Japan

**DOI:** 10.1007/s00431-024-05883-y

**Published:** 2024-11-18

**Authors:** Naomi Matsumoto, Takashi Mitsui, Tomoka Kadowaki, Toshiharu Mitsuhashi, Tomoya Hirota, Hisashi Masuyama, Takashi Yorifuji

**Affiliations:** 1 Department of Epidemiology, Faculty of Medicine, Dentistry and Pharmaceutical Sciences, Okayama University, Okayama, Japan; 2 Department of Obstetrics and Gynecology, Okayama University Graduate School of Medicine, Okayama, Japan; 3 Department of Epidemiology, Graduate School of Medicine, Dentistry and Pharmaceutical Sciences, Okayama University, Okayama, Japan; 4 Center for Innovative Clinical Medicine, Okayama University Hospital, Okayama, Japan; 5 Department of Psychiatry and Behavioral Sciences, UCSF Weill Institute for Neurosciences, University of California San Francisco, San Francisco, USA

**Keywords:** In vitro fertilization (IVF), Assisted reproductive technology (ART), Long-term outcome, Development

## Abstract

The aim of this study is to compare long-term health outcomes between IVF-conceived children and non-IVF-conceived children in Japan, in the context of strong recommendation for single embryo transfer. Using data from a nationwide birth cohort linked with perinatal database, this study analyzed 2140 children born in Japan in May 2010. It compared child health and development outcomes up to 9 years of age between IVF-conceived and non-IVF-conceived children (binary exposure). A Poisson regression with robust variance to estimate the risk ratios for the association between IVF and various long-term child health and developmental outcomes. After adjusting for confounding factors, no significant differences were observed between IVF-conceived and naturally conceived children for most outcomes, including hospitalization, obesity, and developmental milestones. IVF-conceived children showed a slightly lower risk of attention problems at 8 years (adjusted Risk Ratio [aRR]: 0.73, 95% CI: 0.53–1.00). In subgroup analyses, IVF-conceived term children and singletons demonstrated reduced risk of cognitive delays at 5.5 years (aRR: 0.31, 95% CI: 0.10–0.96 and aRR: 0.37, 95% CI: 0.14–0.98, respectively).

*Conclusion*: In this Japanese cohort, IVF conception was not associated with adverse long-term health or developmental outcomes. These findings provide reassurance about the safety of IVF, particularly in the context of single embryo transfer policies. Further research is needed to explore specific IVF protocols and subgroups.

**Supplementary Information:**

The online version contains supplementary material available at 10.1007/s00431-024-05883-y.

**Table:** 

**What is known:** *• Long-term health and developmental outcomes of IVF-conceived children remain inconsistent across studies and populations, despite the widespread use of this technology.*	
**What is new:** *• A nationwide population-based cohort study in Japan did not show adverse effects of IVF on long-term child health and development through age 9.* *• These findings provide reassurance about the safety of IVF, while indicating the need for careful monitoring in specific subgroups such as preterm and multiple births.*	

## Introduction

Assisted reproductive technology (ART), particularly in vitro fertilization (IVF), has become a cornerstone of fertility treatment, leading to the birth of approximately 700,000 of children per year worldwide [1]. Despite the widespread adoption of these technologies, the long-term health outcomes for children conceived via IVF remain an area of active investigation and debate.

Previous research has documented some immediate risks associated with IVF, such as preterm birth and low birth weight, which are often linked to the higher incidence of multiple gestations and preterm birth [2]. However, the long-term health and developmental outcomes for IVF-conceived children are less well understood. Some studies suggest potential long-term health risks, including elevated blood pressure, increased fasting glucose levels, and higher body fat composition [3–6]. These findings, however, are not consistent across all studies, and many investigations suffer from limitations such as small sample sizes and lack of long-term follow-up [7].

This inconsistency in findings and methodological limitations represent a significant knowledge gap in our understanding of the long-term effects of IVF on children’s health and development. Moreover, most studies have been conducted in Western populations, leaving a paucity of data from Asian countries, including Japan, where IVF practices and genetic backgrounds may differ [7].

In Japan, approximately 5000 newborns were conceived through in vitro fertilization (IVF) in 2010 [8], making Japanese women among the most significant users of ART globally. Japan was among the early adopters of single-embryo transfer (SET) policy in IVF treatments, with the Japan Society for Reproductive Medicine and the Japan Society of Obstetrics and Gynecology issuing recommendations for SET in 2007 and 2008, respectively. Following these guidelines, SET proportions gradually increased from 52.2% in 2007 to 82.6% in 2012, which led to a notable decrease in the incidence of twin pregnancies and improved perinatal outcomes, such as reduced rates of preterm delivery and low birth weight [9]. Our study cohort, born in 2010, represents a transitional period in this policy implementation. While multiple births remained more common in IVF pregnancies during this period (20% vs 6% in non-IVF pregnancies in this study), these were exclusively twin pregnancies, with no higher-order multiples observed. This suggests that even during this transition, the SET policy was beginning to show effects in reducing higher-order multiple pregnancies and their associated complications.

Therefore, we conducted a large-scale, longitudinal cohort study in this unique Japanese context. Our research examines over 2000 Japanese infants born during a two-week period in 2010, with follow-up data collected until the age of 9. This study allows us to investigate various health and behavioral outcomes, including preschool hospitalization for respiratory and gastrointestinal issues, obesity, and developmental and behavioral problems. The primary aim of this study is to compare long-term health outcomes between IVF-conceived children and non-IVF-conceived children in Japan, in the context of strong recommendation for single embryo transfer.

## Materials and methods

### Study design/setting/cohort participants

This population-based cohort study utilized data from the Longitudinal Survey of Babies in the 21st Century, conducted by the Ministry of Health, Labour and Welfare (MHLW) of Japan. The survey encompasses one twenty-fourth (1/24) of all births in Japan in 2010, making it well representative of all births in Japan [10]. In the study, we included all children born in Japan between May 10 and 24, 2010, whose perinatal information was successfully linked to the Perinatal Research Network (PRN) database of the Japan Society of Obstetrics and Gynecology (JSOG).

The Longitudinal Survey of Babies in the 21st Century was originally a national initiative to gather information to address the declining birth rate in Japan. The questionnaires were administered in Japanese. This nationwide longitudinal survey covered a wide range of topics, including children's physical development medical history, parental employment and education, smoking history, and thoughts and concerns about parenting, and it has been used in various published studies examining child health outcomes [11–14] and developmental outcomes [15, 16]. The MHLW mailed baseline questionnaires to parents of all 43,767 children (including multiple births) when the children were six months of age. The response rate for the baseline survey was 88.1% (38,554 children). The children’s guardians who responded to the baseline questionnaire received follow-up questionnaires every year until the children reached 5.5 years of age, and then annually at 7, 8, and 9 years of age. Cohort questionnaires covered a wide range of topics, including children’s physical development [11, 12], medical history [13, 14], parental employment and education, smoking history, and thoughts and concerns about parenting. Birth record data from Japan’s vital statistics were also linked to each participant.

The PRN database is a nationwide registry of births and stillbirths after 22 weeks gestation, which was initiated by JSOG in the early 2000s. Participating facilities mainly include maternal and child centers that deal with high-risk pregnancies. Obstetricians record information on maternal characteristics, pre-existing conditions, pregnancy complications, delivery details, and neonatal transfers in a preset format. In 2010, the PRN included data on 83,383 children from 139 facilities, representing 7.6% of all births in Japan that year [17, 18]. In 2010, approximately 2.7% of all births in Japan were attributed to IVF [8]. However, the PRN database showed that 11.4% (9511) of the 83,383 registered children were conceived through infertility treatment, including 4342 (5.2%) by IVF [17].

**Fig. 1:**
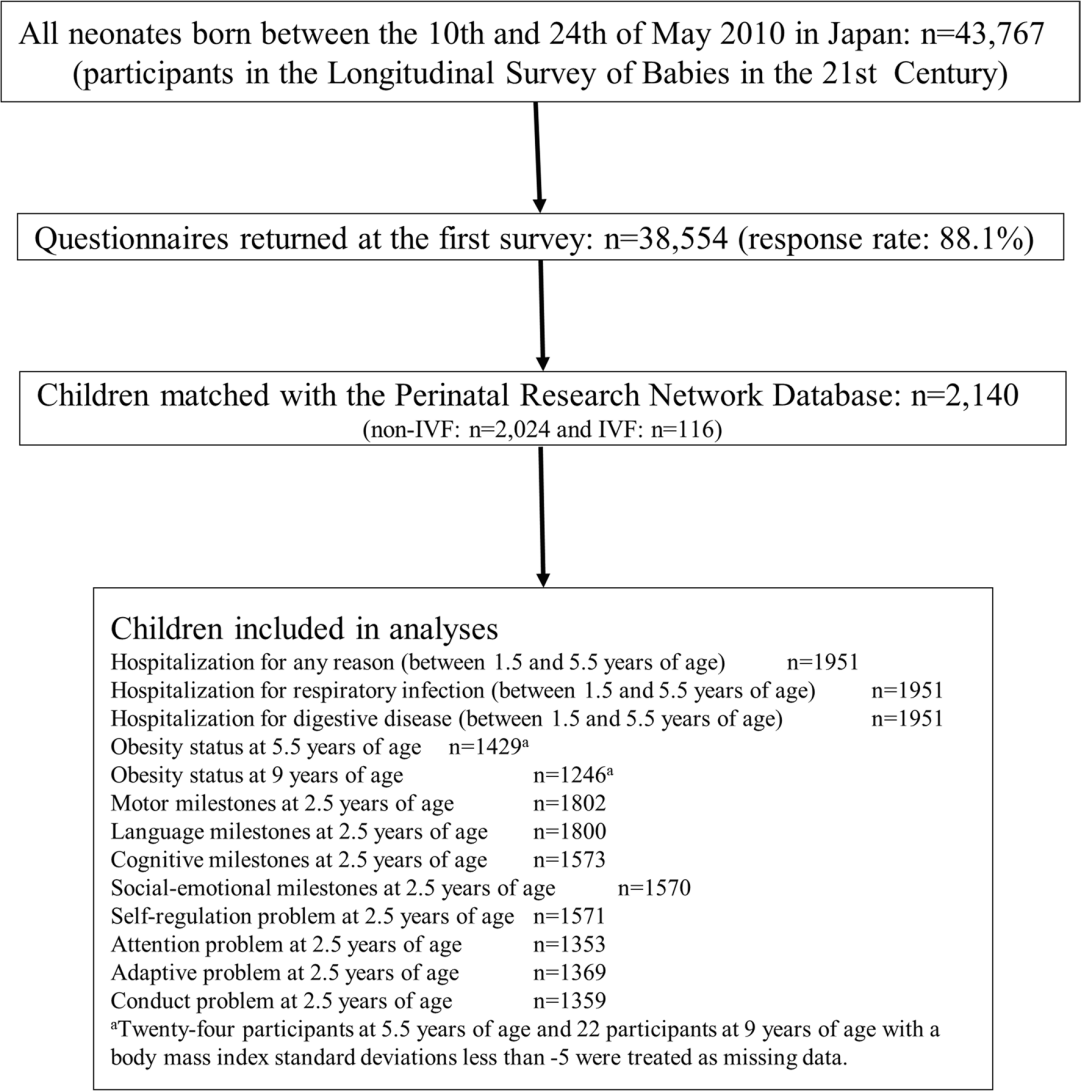
Flowchart of the participants. Numbers in boxes represent the number of children with available follow-up data for each outcome, not the number of children who experienced the outcome. More detailed outcome data are presented in Table 2

To identify study participants, we linked the PRN data to the Longitudinal Study of Babies in the 21st Century (2010 cohort) using the child’s date of birth, sex, birth weight, maternal age at delivery, gestational weeks, and gestational days. A total of 2140 children born in May 2010 who could be linked were included in the analysis (Fig. 1).

### Exposure: information on IVF

The PRN database is primarily completed by obstetricians and/or midwives and includes a section inquiring about infertility treatment. There are five options: “none”, “ovulation induction”, “AIH (Artificial Insemination by Husband)”, “IVF-ET (In Vitro Fertilization and Embryo Transfer)”, and “others”, with multiple choices possible. Respondents who selected IVF-ET were defined as the IVF group, while those who did not select IVF-ET were defined as the non-IVF group (binary exposure). In other words, it should be noted that in this study, IVF referred to all procedures related to ART, including not only IVF itself but also other techniques such as intracytoplasmic sperm injection (ICSI) and egg donation.

### Outcomes: information on child health outcomes

#### Preschool hospitalizations

In the Longitudinal Survey of Babies in the 21st Century, respondents were annually asked whether their children had been hospitalized for treatment in the past year and the cause of the hospitalization. Using information from the second through sixth surveys (aged 1.5–5.5 years), we defined the preschool hospitalization group as those who had at least one inpatient hospitalization for any reason. Similarly, children who were hospitalized at least once during the same period for respiratory tract infections were defined as the preschool respiratory infection hospitalization group. Additionally, those who were hospitalized for gastroenteritis or other gastrointestinal tract illnesses (excluding intestinal intussusception) were defined as the preschool gastrointestinal tract illness hospitalization group. The frequency of hospitalizations during each period was not collected in this survey.

#### Anthropometric measurements

The body mass index (BMI) standard deviation score (SD score) was calculated from height and weight at 5.5 and 9 years of age, and defined as follows: less than − 1.5 SD as underweight, − 1.5 SD to 1.5 SD as normal weight, 1.5 SD to 2.5 SD as overweight, and 2.5 SD or more as obese according to World Health Organization criteria. To address the potential for outliers, we excluded children with BMI SD scores less than − 5 SD at 5.5 years of age (*n* = 4) and 9 years of age (*n* = 2), all of whom were non-IVF. In this study, those who were overweight or obese were defined as obese.

#### Developmental milestones

The Longitudinal Survey of Babies in the 21st Century involved a variety of developmental assessments of children at different ages [19]. Binary responses (“Yes” and “No”) were used in all questions pertaining to child development in the survey. Two experts (a board-certified pediatrician [NM] and a board-certified child psychiatrist specializing in child development [TH]) carefully reviewed each item in the survey. They categorized the items into the following developmental domains (eTable 1), and defined those who answered “No” to even one item in each domain as not having achieved development in that domain: motor milestone (4 items) at 2.5 years of age, language milestone (3 items) at 2.5 years of age, cognitive milestone (1 item) at 5.5 years of age, self-regulation (2 items) at 5.5 years of age, social-emotional milestone (3 items) at 5.5 years of age, attention (1 item) at 8 years old, adaptive skills (3 items) at 8 years of age, and conduct problems (3 items) at 8 years of age.

These developmental domains for survey items were defined by a consensus of the 2 authors mentioned above based on items of the milestone assessments of the Centers for Disease Control and Prevention (CDC) [20], the Behavioral Assessment System for Children, Third Edition (BASC3) [21], and the Strengths and Difficulties Questionnaire (SDQ) [22–24]. The definitions of developmental domains and items included in each domain were reviewed and approved by another pediatric physician (TY).

More details on the specific assessment items of each milestone are shown in eTable 1.

### Statistical analysis

We compared parental and neonatal characteristics between IVF and non-IVF groups. As this study had multiple outcome time points and measures, we compared characteristics of the dropout and non-dropout (analysis) groups, focusing on obesity at 9 years, the longest follow-up outcome, to consider potential attrition bias. Summaries for categorical variables are presented as counts (percentages), while continuous variables are summarized as means (standard deviations) when normally distributed and as median (interquartile range) when not. The normality of continuous variables was assessed using Shapiro–Wilk tests and visual inspection of histograms and distribution plots.

We then conducted a Poisson regression with robust variance to estimate the risk ratios for the association between IVF and various long-term child health and developmental outcomes, such as hospitalization, obesity and developmental milestones. Following a crude analysis (model 1), we conducted a controlled analysis (model 2: complete case analysis) to adjust for confounding variables. The adjustment variables were selected based on previous studies [3, 25] and domain knowledge as follows: maternal age at delivery (< 30, 30–34, and ≥ 35 years; categorical), parity (binary), maternal pre-existing conditions (binary), maternal smoking during pregnancy (binary), maternal alcohol consumption during pregnancy (binary), maternal education attainment (bachelor’s degree or higher, vocational school/junior college graduate, and high school graduate or lower; categorical), paternal age at delivery (same as maternal; categorical), paternal education attainment (same as maternal; categorical), and place of residence at birth (special ward or designated city, city, and town or village; categorical). In the obesity outcome model, maternal pre-pregnancy BMI (continuous) was also added as an adjustment variable. In model 3, missing data of covariates were dealt with by multiple imputation with the method of chained equations. The percentage of missing data ranged from 0 to 26.4%. Multiple imputation was performed using 400 iterations with 20 burn-ins. The following covariates were used for multiple imputation: maternal age at delivery (no missing data), parity (missing data: 0.7%), maternal pre-pregnancy BMI (missing data: 16.0%), maternal smoking during pregnancy (missing data: 26.3%), maternal alcohol consumption during pregnancy (missing data: 26.4%), maternal pre-existing conditions (no missing data), maternal education (missing data: 12.8%), paternal age (missing data: 2.6%), paternal education (missing data: 14.2%), and place of residence at birth (no missing data). In addition, subgroup analyses were performed stratified by the presence or absence of preterm birth and multiple births.

All analyses were performed with STATA SE version 18 (StataCorp., College Station, TX, USA). For all analyses, we adopted 5% as significant level.

### Ethical approval

This study was approved by the Institutional Review Board of the Graduate School of Biomedical Sciences, Okayama University (No. 2310–018), and the Clinical Research Management and Review Committee of the Japan Society of Obstetrics and Gynecology (No. 150).

## Results

### Demographic characteristics

The baseline characteristics of the parents with and without IVF and their children are shown in Table 1. Children born through IVF were more likely to have older parents, have primiparous mothers, be born preterm, have a low birth weight, be small for gestational age, have multiple births, be born by cesarean section, and be in breech presentation than non-IVF-conceived children. Maternal pre-existing conditions and pregnancy complications were more common in IVF-conceived children. Specifically, the proportions of reproductive organ or thyroid disease as maternal pre-existing conditions and those of threatened abortion or preterm labor, placenta previa, or premature rupture of membranes as pregnancy complications were higher in IVF-conceived children than in non-IVF-conceived children. The percentages of children with low parental education and living in a town or village were lower in IVF-conceived children than in IVF-conceived children. Mothers in the dropout group were younger, had lower education levels, and had a higher prevalence of smoking during pregnancy than mothers in the non-dropout group (eTable 2).

**Table 1:** Demographics of the participants

	Non-IVF	IVF	Total
	(*n* = 2024)	(*n* = 116)	(*n* = 2140)
Gestational week*	38 (3)	38 (2)	38 (3)
Birth weight (g)*	2890.5 (621.0)	2858.0 (738.5)	2888.5 (627.0)
Preterm birth < 37 weeks	303 (15.0%)	25 (21.6%)	328 (15.3%)
Low birth weight < 2500 g	449 (22.2%)	35 (30.2%)	484 (22.6%)
Small for gestational age	120 (5.9%)	9 (7.8%)	129 (6.0%)
Cesarean section	699 (35.0%)	64 (55.2%)	763 (36.1%)
Position			
Cephalic presentation	1879 (92.8%)	101 (87.1%)	1980 (92.5%)
Breech presentation	129 (6.4%)	13 (11.2%)	142 (6.6%)
Others	16 (0.8%)	2 (1.7%)	18 (0.8%)
Multiple births	128 (6.3%)	24 (20.7%)	152 (7.1%)
Parity			
Primipara	1043 (51.9%)	86 (74.1%)	1129 (53.1%)
Multipara	966 (48.1%)	30 (25.9%)	996 (46.9%)
Neonatal asphyxia			
No	1942 (96.6%)	114 (98.3%)	2056 (96.7%)
Mild neonatal asphyxia	61 (3.0%)	2 (1.7%)	63 (3.0%)
Severe neonatal asphyxia	8 (0.4%)	0 (0.0%)	8 (0.4%)
Maternal pre-existing conditions	626 (30.9%)	46 (39.7%)	672 (31.4%)
Diseases of the reproductive organs	202 (10.0%)	18 (15.5%)	220 (10.3%)
Thyroid diseases	58 (2.9%)	8 (6.9%)	66 (3.1%)
Hypertension	24 (1.2%)	0 (0.0%)	24 (1.1%)
Diabetes (including gestational diabetes mellitus)	52 (2.6%)	2 (1.7%)	54 (2.5%)
Pregnancy complications	1167 (57.7%)	82 (70.7%)	1249 (58.4%)
Threatened abortion	28 (1.4%)	7 (6.0%)	35 (1.6%)
Threatened preterm labor	325 (16.1%)	25 (21.6%)	350 (16.4%)
Placenta previa	38 (1.9%)	4 (3.4%)	42 (2.0%)
Premature rupture of membranes	281 (13.9%)	21 (18.1%)	302 (14.1%)
Prolonged labor	40 (2.0%)	4 (3.4%)	44 (2.1%)
Fetal distress	96 (4.7%)	7 (6.0%)	103 (4.8%)
Placental adhesion	6 (0.3%)	3 (2.6%)	9 (0.4%)
Preeclampsia	124 (6.1%)	10 (8.6%)	134 (6.3%)
Maternal smoking during pregnancy	80 (5.4%)	1 (1.2%)	81 (5.1%)
Maternal alcohol consumption during pregnancy	58 (3.9%)	3 (3.6%)	61 (3.9%)
Maternal age at delivery (years)			
< 30	642 (31.7%)	3 (2.6%)	645 (30.1%)
30–34	730 (36.1%)	32 (27.6%)	762 (35.6%)
35 +	652 (32.2%)	81 (69.8%)	733 (34.3%)
Paternal age at delivery			
< 30	475 (24.1%)	3 (2.6%)	478 (22.9%)
30–34	622 (31.6%)	14 (12.1%)	636 (30.5%)
35 +	872 (44.3%)	99 (85.3%)	971 (46.6%)
Maternal education attainment			
Bachelor’s degree or higher	533 (30.3%)	36 (33.0%)	569 (30.5%)
Vocational school/junior college graduate	745 (42.4%)	58 (53.2%)	803 (43.0%)
High school graduate or lower	480 (27.3%)	15 (13.8%)	495 (26.5%)
Paternal education attainment			
Bachelor’s degree or higher	917 (53.1%)	65 (59.6%)	982 (53.5%)
Vocational school/junior college graduate	275 (15.9%)	16 (14.7%)	291 (15.8%)
High school graduate or lower	536 (31.0%)	28 (25.7%)	564 (30.7%)
Place of residence at birth			
Special ward or designated city	860 (42.5%)	55 (47.4%)	915 (42.8%)
City	1040 (51.4%)	60 (51.7%)	1100 (51.4%)
Town or village	124 (6.1%)	1 (0.9%)	125 (5.8%)

* Gestational week and birth weight displayed as Median (IQR)

### IVF and long-term child health and developmental outcomes

We observed no statistically significant differences between IVF-conceived children and non-IVF children for most of the outcomes studied (Table 2). This includes hospitalization rates, weight status at different ages, and various developmental milestones.

**Table 2:** In vitro fertilization effects on child outcomes

	Crude model		Adjusted modela	Multiple imputation adjusted modela
	n/N (%)	Crude RR (95%CI)	aRR (95%CI) 95% CI	aRR (95%CI)
Health outcomes				
Hospitalization for any reason				
IVF	28/111 (25.2%)	1.06 (0.76, 1.48) 0.098	1.20 (0.79, 1.82) 0.149	1.16 (0.82, 1.64)−0.054 0.126
Non-IVF	436/1,840 (23.7%)	1 (reference)	1 (reference)	1 (reference)
Hospitalization for respiratory infection				
IVF	11/111 (9.9%)	0.99 (0.55, 1.76) 0.056	1.42 (0.73, 2.76)	1.22 (0.67, 2.22)−0.046
Non-IVF	185/1,840 (10.1%)	1 (reference)	1 (reference)	1 (reference)
Hospitalization for gastrointestinal disease				
IVF	1/111 (0.9%)	0.29 (0.04, 2.08) −0.003	0.34 (0.05, 2.41)	0.28 (0.04, 1.96)−0.039-0.006
Non-IVF	57/1,840 (3.1%)	1 (reference)	1 (reference)	1 (reference)
Overweight or obesity at 5.5 years of age				
IVF	4/90 (4.4%)	0.45 (0.17, 1.18)−0.010	0.28 (0.07, 1.09)	0.44 (0.16, 1.16)−0.099-0.011
Non-IVF	133/1,399 (9.9%)	1 (reference)	1 (reference)	1 (reference)
Overweight or obesity at 9 years of age				
IVF	11/79 (13.9%)	1.14 (0.65, 2.02) 0.098	0.90 (0.38, 2.15)	1.31 (0.73, 2.36)
Non-IVF	142/1,167 (12.2%)	1 (reference)	1 (reference)	1 (reference)
Developmental outcomes				
Motor milestones not attained by the expected time (2.5 years)				
IVF	9/107 (8.4%) 0.13 (0.52)	1.64 (0.85, 3.16)	1.65 (0.77, 3.52)	1.18 (0.59, 2.36)
Non-IVF	87/1,608 (5.1%) 0.09 (0.48)	1 (reference)	1 (reference)	1 (reference)
Language milestones not attained by the expected time (2.5 years)				
IVF	17/107 (15.9%) 0.26 (0.68)	0.96 (0.61, 1.50)	0.94 (0.55, 1.61)	0.85 (0.54, 1.34)
Non-IVF	281/1,693 (16.6%) 0.26 (0.65)	1 (reference)	1 (reference)	1 (reference)
Cognitive milestones not attained by the expected time (5.5 years)				
IVF	6/96 (6.3%) 0.06 (0.24)	0.74 (0.34, 1.65)	0.62 (0.23, 1.69)	0.57 (0.25, 1.27)
Non-IVF	124/1,477 (8.4%) 0.08 (0.28)	1 (reference)	1 (reference)	1 (reference)
Social-emotional milestones not attained by the expected time (5.5 years)				
IVF	14/96 (14.6%) 0.18 (0.46)	0.98 (0.60, 1.62) 0.098	0.82 (0.45, 1.50)	0.89 (0.53, 1.48)
Non-IVF	219/1,475 (14.9%) 0.21 (0.56)	1 (reference)	1 (reference)	1 (reference)
Self-regulation problem (5.5 years)				
IVF	10/96 (10.4%) 0.11 (0.35)	0.80 (0.44, 1.47) 0.098	0.60 (0.27, 1.32)	0.70 (0.38, 1.27)
Non-IVF	191/1,474 (13.0%) 0.16 (0.44)	1 (reference)	1 (reference)	1 (reference)
Attention problem (8 years)				
IVF	28/92 (30.4%) 0.21 (0.41)	0.81 (0.59, 1.12) 0.098	0.73 (0.51, 1.06)	0.73 (0.53, 1.00)
Non-IVF	475/1,277 (37.4%) 0.21 (0.41)	1 (reference)	1 (reference)	1 (reference)
Adaptive problem (8 years)				
IVF	34/92 (37.0%) 0.48 (0.72)	1.11 (0.84, 1.47) 0.098	1.13 (0.81, 1.56)	1.01 (0.76, 1.34)
Non-IVF	419/1,261 (33.2%) 0.41 (0.65)	1 (reference)	1 (reference)	1 (reference)
Conduct problem (8 years)				
IVF	29/92 (31.5%) 0.42 (0.71)	0.94 (0.69, 1.29) 0.098	0.98 (0.67, 1.43)	0.97 (0.70, 1.34)
Non-IVF	423/1,267 (33.4%) 0.46 (0.74)	1 (reference)	1 (reference)	1 (reference)

RR: risk ratio, aRR: adjusted risk ratio, CI: confidence interval

^a^Adjusted for maternal age at delivery, parity, maternal underlying diseases, pregnancy complications, maternal smoking during pregnancy, maternal alcohol consumption during pregnancy, maternal education attainment, paternal age, paternal education attainment, and place of residence at birth. In the obesity outcome model, maternal pre-pregnancy BMI (continuous) was also added as an adjustment variable.

*RR*, risk ratio; *aRR*, adjusted risk ratio; *CI*, confidence interval

^a^Adjusted for maternal age at delivery, parity, maternal underlying diseases, pregnancy complications, maternal smoking during pregnancy, maternal alcohol consumption during pregnancy, maternal education attainment, paternal age, paternal education attainment, and place of residence at birth. In the obesity outcome model, maternal pre-pregnancy BMI (continuous) was also added as an adjustment variable

The only notable finding that approached statistical significance in the main analysis was related to attention problems at 8 years of age, where IVF children showed a slightly lower risk (adjusted Risk Ratio [aRR]: 0.73, 95% CI: 0.53–1.00) compared to non-IVF children.

### Subgroup analysis by preterm and multiple births

**Fig. 2:**
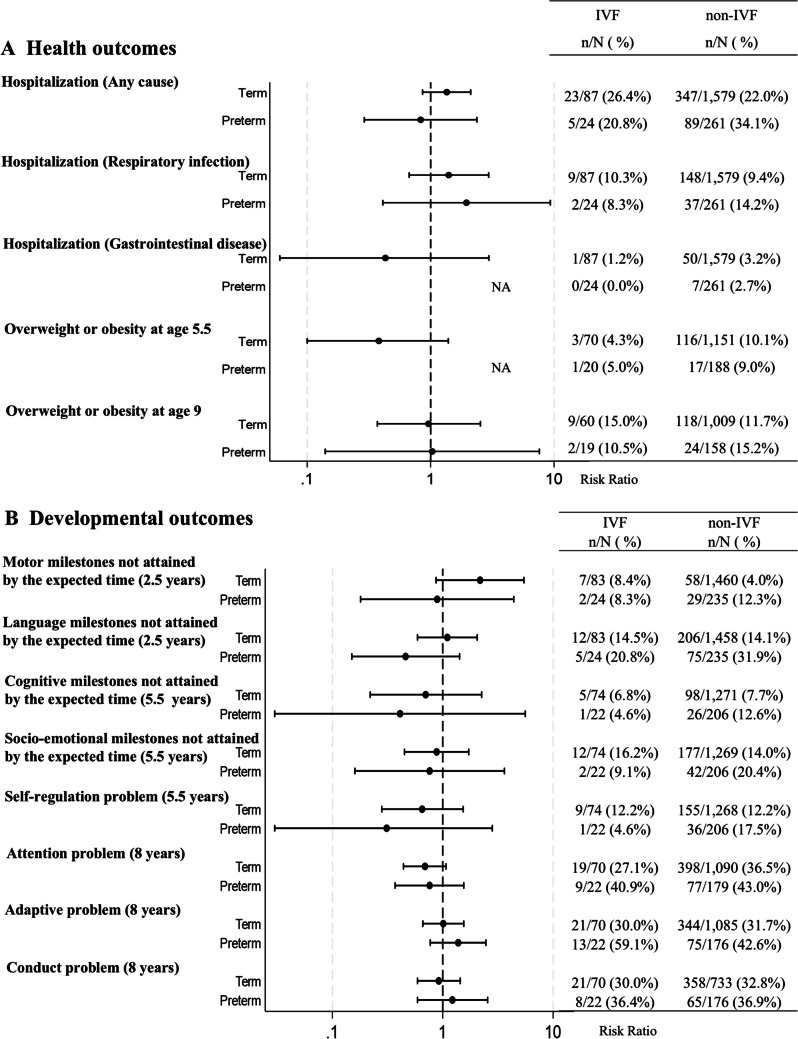
Associations of IVF on child health outcomes (adjusted model). The complete case analysis model was adjusted for maternal age at delivery, maternal underlying diseases, pregnancy complications, maternal smoking during pregnancy, maternal alcohol consumption during pregnancy, maternal education, paternal age, paternal education, and place of residence at birth. In the obesity outcome model, maternal pre-pregnancy body mass index was also adjusted for. NA: not applicable

IVF-conceived term children exhibited a reduced risk of failing to attain cognitive milestones by the expected time at 5.5 years (adjusted relative risk [aRR]: 0.31, 95% confidence interval [CI]: 0.10–0.96). IVF-conceived preterm children demonstrated higher risk of all-cause hospitalization (aRR: 2.03, 95% CI: 1.14–3.61) but lower risk of attention problems at 8 years (aRR: 0.46, 95% CI: 0.25–0.86) (Fig. 2).

**Fig. 3:**
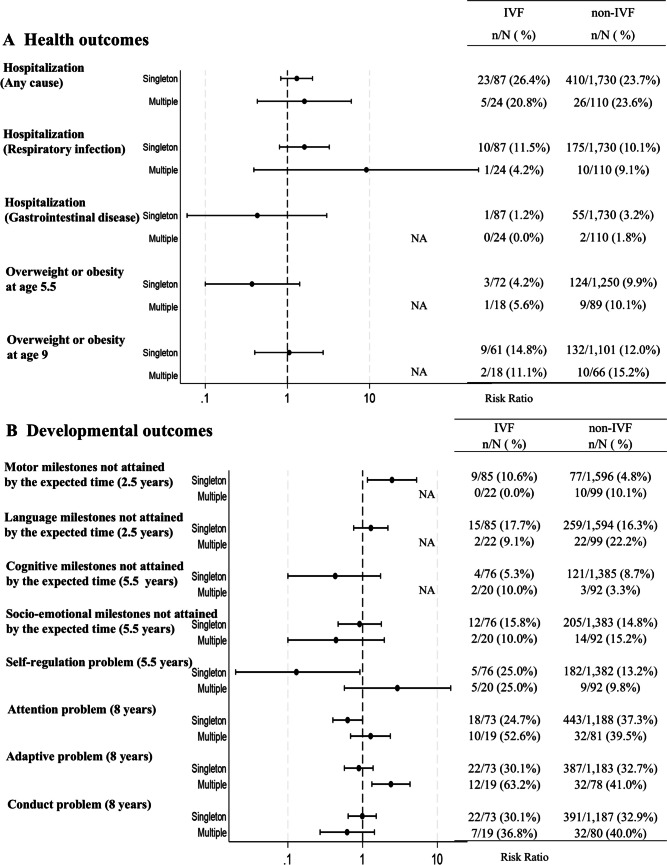
Associations of IVF on child developmental outcomes (adjusted model). The model was adjusted for maternal age at delivery, maternal underlying diseases, pregnancy complications, maternal smoking during pregnancy, maternal alcohol consumption during pregnancy, maternal education, paternal age, paternal education, and place of residence at birth. NA: not applicable

Similarly, IVF-conceived singletons showed a decreased risk of failing to attain cognitive milestones by the expected time at 5.5 years (aRR: 0.37, 95% CI: 0.14–0.98). IVF-conceived multiples exhibited a higher risk of all-cause hospitalization (aRR: 1.94, 95% CI: 1.12–3.37) but a lower risk of attention problems at 8 years (aRR: 0.42, 95% CI: 0.24–0.74) (Fig. 3).

## Discussion

Using data from the 2010 Japanese nationwide birth cohort, this study examined the association between IVF and child health and developmental outcomes. Although children conceived through IVF have a slightly higher risk of some perinatal and maternal complications, the effects of IVF on most of the child health and developmental outcomes examined were non-adverse. IVF was even potentially protective against attention problems at 8 years of age. IVF-conceived term children and IVF-conceive singletons had a reduced risk of cognitive delays at 5.5 years. IVF-conceived preterm children and IVF- conceived multiples had a higher risk of all-cause hospitalization but a lower risk of attention problems at 8 years.

Previous studies’ findings on IVF’s long-term impact on children have been inconsistent. Some found no association between ART and IVF on child developmental outcomes [25, 26], while others reported protective effects for autism spectrum disorder, attention deficit hyperactivity disorder, and poor school performance [27], or increased risk for autism spectrum disorder and other developmental/intellectual disabilities [28–30]. Notably, these discrepancies were often reported to attenuate when adjusting for preterm or multiple births.

Our study suggests that IVF does not directly increase the likelihood of unfavorable child health and developmental outcomes. This finding has several important implications. First, regarding physical health outcomes, we found no increased risks of hospitalization or obesity among IVF-conceived children in general, though subgroup analyses revealed higher hospitalization risks in specific groups (preterm and multiple births). This suggests that IVF itself may not compromise general physical health, but certain subgroups may need closer monitoring. Second, in terms of developmental outcomes, we found no delays in attaining developmental milestones or increased challenges with self-regulation and conduct. This is particularly reassuring given previous concerns about potential developmental impacts of assisted reproductive technologies. The observed protective association with attention problems is especially noteworthy. This finding might reflect various underlying factors. Parents of IVF-conceived children often show heightened awareness and engagement in their child's development, potentially leading to more attentive monitoring and support. These families typically demonstrate better healthcare service utilization patterns, including regular check-ups and preventive care. Additionally, there may be earlier recognition and intervention for developmental concerns in this population. The careful embryo selection and culture conditions in IVF procedures might also play a role through biological mechanisms that warrant further investigation.

Our research utilized a large, nationwide sample with a longitudinal design extending through multiple developmental periods. Strengths of this study include its follow-up to 9 years of age, linking of data from a nationwide birth cohort in Japan to a perinatal database composed mainly of maternal and child health centers with a large number of births from infertility treatment. We conducted comprehensive assessments across multiple health and developmental domains, employing robust statistical analyses to address potential confounding factors. Our findings contribute to the growing body of evidence supporting the long-term safety of IVF, while also highlighting areas requiring continued attention and monitoring. The results are particularly meaningful given our study's methodological strengths.

However, this study has several limitations. First, this study included only Japanese children (*n* = 2140). The generalizability of our findings should be considered within Japan's universal health insurance system, which ensures consistent access to medical care and standardized fertility treatment protocols. While the original cohort was representative of births in Japan as a whole, the study's generalizability is possibly limited because it only included children who were linked to a perinatal database primarily covering high-risk pregnancies. However, no notable differences in outcome proportions were observed between the analysis group and the original cohort, (eTable 3), suggesting our findings may be generalizable to the broader Japanese population. Second, potential selection bias exists due to loss to follow-up, with the dropout group having mothers with lower educational attainment and higher smoking proportions. Third, our non-IVF group included children conceived through fertility treatments other than IVF (such as ovulation induction and artificial insemination by husband [AIH]), which might have attenuated between-group differences. Fourth, child development and health outcomes relied solely on parental reports through surveys. While this survey has been previously utilized in multiple studies [11–16], parent-reported data may be subject to reporting biases that could differ between IVF and non-IVF parents. Moreover, the developmental assessment was based on binary responses without validated scales, although the domains were defined based on established assessments (CDC, BASC3, and SDQ). Future studies incorporating objective clinical assessments would be valuable to validate these findings. Fifth, despite adjusting for socioeconomic factors, the comparability of groups remains a concern, though, Japan’s IVF subsidy program for households below the income limit is subsidized up to twice a year, starting in 2004 at 100,000 yen per transplant and expanding to 150,000 yen per transplant in 2009, may help mitigate cost-related selection bias. There is also a limitation regarding our statistical approach to missing data. Some variables, particularly maternal smoking (26.3%) and alcohol consumption during pregnancy (26.4%), had high proportions of missing data. Therefore, in addition to complete case analysis (under Missing Completely At Random assumption), we performed multiple imputation (under Missing At Random assumption), but we did not account for potential Missing Not At Random mechanisms [31]. Finally, we could not examine certain important outcomes (e.g., cardiac problems) or specific IVF procedures (e.g., ICSI), which warrant investigation in future studies given their potential associations with developmental outcomes [32].

## Conclusions

This Japanese nationwide cohort study, particularly in the context of strong recommendation for single embryo transfer, did not observe an association between IVF and the long-term health and developmental outcomes of children. Our findings provide encouraging evidence for the overall safety of IVF treatment, and they add to the ongoing discussion on the long-term effects of IVF-conceived children. Future research should focus on specific IVF protocols and subgroups, such as multiple births and paternal infertility, to further understand the potential risks and benefits of IVF.

## Supplementary Information

Below is the link to the electronic supplementary material.

Supplementary file1 (DOCX 37 KB)

## Data Availability

De-identified individual participant data will be made available upon reasonable request and approval by the Ministry of Health, Labour and Welfare and the Japan Society of Obstetrics and Gynecology.
