# Supplementary material for: In vitro fertilization and long-term child health and development: nationwide birth cohort study in Japan
Source: Eur J Pediatr. 2024 Nov 18;184(1):24. doi: 10.1007/s00431-024-05883-y (PMC11573831; doi:10.1007/s00431-024-05883-y)
Supplement: Supplementary file 1 — Supplementary file1 (DOCX 37 KB) [file 431_2024_5883_MOESM1_ESM.docx]

| eTable 1. Questionnaires for Assessing Developmental Milestones and Behavioral Indicators | | | |
| --- | --- | --- | --- |
|  | Questionnaires | Developmental milestones | Reference |
| The following items were selected as "yes" or "no" in the survey at 2.5 years of age | | |  |
|  | Can your child walk? | Motor milestone | CDC |
|  | Can your child run? | Motor milestone | CDC |
|  | Can your child climb stairs? | Motor milestone | CDC |
|  | Can your child use a spoon (fork) for self-feeding? | Motor milestone | CDC |
|  | Can your child say words with meaning? | Language milestone | Expert opinion referred to CDC |
|  | Can your child compose two-phrase sentences? | Language milestone | CDC |
|  | Can your child say his or her own name? | Language milestone | CDC |
| The following items were selected as "can" or "cannot" in the survey at 5.5 years of age | | | |
|  | Focusing on one thing | Cognitive milestones | CDC |
|  | Expressing emotions appropriately | Social-emotional milestones | Expert opinion |
|  | Acting in a group | Social-emotional milestones | Expert opinion |
|  | Keeping promises | Social-emotional milestones | CDC |
|  | Listening calmly | Self-regulation | Expert opinion |
|  | Enduring or tolerating | Self-regulation | Expert opinion |
| The following behavioral indicators were selected as "has acquired" or "has not acquired" in the survey at 8 years of age | | | |
|  | Listens to the end of what others say | Attention problem | BASC3 (PRS) |
|  | Greets and responds to others | Adaptive skills | BASC3 (PRS) |
|  | Always looks right and left when crossing the road | Adaptive skills | BASC3 (PRS) |
|  | Does not follow strangers | Adaptive skills | BASC3 (PRS) |
|  | Does not tell a lie | Conduct problem | SDQ_18/BASC3 (PRS) |
|  | Does not break toys or books | Conduct problem | BASC3/CBCL |
|  | Is not violent to others | Conduct problem | SDQ_18/BASC3 (PRS) |
| CDC: Centers for Disease Control and Prevention, | |  |  |
| BASC3 (PRS): Behavior Assessment System for Children, Third Edition, Parent Rating Scales, | | | |
| SDQ: Strengths and Difficulties Questionnaire, | |  |  |
| CBCL: Child Behavior Checklist. | |  |  |

| eTable 2. Characteristics of Participants Who Were Lost to Follow-up | | | |
| --- | --- | --- | --- |
|  | Analyzed | Lost to follow up | Total |
|  | (n=1246) | (n=894) | (n=2140) |
| *In vitro* fertilization | 79 (6.3) | 37 (4.1) | 116 (5.4) |
| Gestational week* | 38 (3) | 38 (2) | 38 (3) |
| Birth weight (g)* | 2892.0 (619.0) | 22885.0 (636.0) | 2888.5 (627.0) |
| Preterm birth <37 weeks | 177 (14.2%) | 151 (16.9%) | 328 (15.3%) |
| Low birth weight < 2500 g | 275 (22.1%) | 209 (23.4%) | 484 (22.6%) |
| Small for gestational age | 75 (6.0%) | 54 (6.0%) | 129 (6.0%) |
| Cesarean section | 450 (36.5%) | 313 (35.5%) | 763 (36.1%) |
| Fetal presentation |  |  |  |
| Cephalic presentation | 1147 (92.1%) | 833 (93.2%) | 1980 (92.5%) |
| Breech presentation | 88 (7.1%) | 54 (6.0%) | 142 (6.6%) |
| Others | 11 (0.9%) | 7 (0.8%) | 18 (0.8%) |
| Multiple births | 84 (6.7%) | 68 (7.6%) | 152 (7.1%) |
| Parity |  |  |  |
| Primipara | 661 (53.3%) | 468 (52.8%) | 1,129 (53.1%) |
| Multipara | 578 (46.7%) | 418 (47.2%) | 996 (46.9%) |
| Neonatal asphyxia |  |  |  |
| No | 1197 (96.7%) | 859 (96.6%) | 2056 (96.7%) |
| Mild neonatal asphyxia | 38 (3.1%) | 25 (2.8%) | 63 (3.0%) |
| Severe neonatal asphyxia | 3 (0.2%) | 5 (0.6%) | 8 (0.4%) |
| Maternal pre-existing conditions | 398 (31.9%) | 274 (30.6%) | 672 (31.4%) |
| Pregnancy complications | 741 (59.5%) | 508 (56.8%) | 1,249 (58.4%) |
| Maternal smoking during pregnancy | 24 (2.6%) | 57 (8.8%) | 81 (5.1%) |
| Maternal alcohol consumption during pregnancy | 35 (3.8%) | 26 (4.0%) | 61 (3.9%) |
| Maternal age at delivery (years) |  |  |  |
| <30 | 299 (24.0%) | 346 (38.7%) | 645 (30.1%) |
| 30-34 | 455 (36.5%) | 307 (34.3%) | 762 (35.6%) |
| 35+ | 492 (39.5%) | 241 (27.0%) | 733 (34.3%) |
| Paternal age at delivery (years) |  |  |  |
| <30 | 229 (18.5%) | 249 (29.3%) | 478 (22.9%) |
| 30-34 | 378 (30.6%) | 258 (30.4%) | 636 (30.5%) |
| 35+ | 629 (50.9%) | 342 (40.3%) | 971 (46.6%) |
| Maternal education attainment |  |  |  |
| Bachelor's degree or higher | 402 (33.2%) | 167 (25.5%) | 569 (30.5%) |
| Vocational school/junior college graduate | 531 (43.8%) | 272 (41.5%) | 803 (43.0%) |
| High school graduate or lower | 279 (23.0%) | 216 (33.0%) | 495 (26.5%) |
| Paternal education attainment |  |  |  |
| Bachelor's degree or higher | 695 (57.8%) | 287 (45.2%) | 982 (53.5%) |
| Vocational school/junior college graduate | 179 (14.9%) | 112 (17.6%) | 291 (15.8%) |
| High school graduate or lower | 328 (27.3%) | 236 (37.2%) | 564 (30.7%) |
| Place of residence at birth |  |  |  |
| Special ward or designated city | 536 (43.0%) | 379 (42.4%) | 915 (42.8%) |
| City | 647 (51.9%) | 453 (50.7%) | 1100 (51.4%) |
| Town or village | 63 (5.1%) | 62 (6.9%) | 125 (5.8%) |
| * Gestational week and birth weight are displayed as Median (IQR)  Other items are displayed as n (%) | | | |

| eTable 3. Proportions of Each Outcome among Children Analyzed and All Children with mothers aged over 25 years included in 2010 Cohort | | | | | | |
| --- | --- | --- | --- | --- | --- | --- |
|  |  |  | Analyzed | | Entire 2010 Cohort | |
| Hospitalization for any reason | Yes | | 23.8 | % | 21.2 | % |
| Hospitalization for respiratory infection | Yes | | 10.1 | % | 8.8 | % |
| Hospitalization for gastrointestinal disease | Yes | | 3.0 | % | 2.9 | % |
| Overweight or obesity at age 5.5 years | Yes | | 9.6 | % | 10.0 | % |
| Overweight or obesity at age 9 years | Yes | | 12.3 | % | 13.1 | % |
|  |  |  |  |  |  |  |
| Motor milestones not attained by the expected time (2.5 years) | Score | 0 | 94.7 | % | 96.8 | % |
|  |  | 1 | 3.3 | % | 2.5 | % |
|  |  | 2 | 0.6 | % | 0.3 | % |
|  |  | 3 | 0.7 | % | 0.2 | % |
|  |  | 4 | 0.8 | % | 0.2 | % |
| Language milestones not attained by the expected time (2.5 years) | Score | 0 | 83.4 | % | 88.2 | % |
|  |  | 1 | 9.3 | % | 8.0 | % |
|  |  | 2 | 5.2 | % | 3.1 | % |
|  |  | 3 | 2.1 | % | 0.7 | % |
| Cognitive milestones not attained by the expected time (5.5 years) | Score | 0 | 91.7 | % | 92.8 | % |
|  |  | 1 | 8.3 | % | 7.2 | % |
| Social-emotional milestones not attained by the expected time (5.5 years) | Score | 0 | 85.2 | % | 87.0 | % |
|  |  | 1 | 10.7 | % | 9.7 | % |
|  |  | 2 | 2.6 | % | 2.4 | % |
|  |  | 3 | 1.6 | % | 0.9 | % |
| Self-regulation problem (5.5 years) | Score | 0 | 87.2 | % | 87.9 | % |
|  |  | 1 | 10.0 | % | 10.2 | % |
|  |  | 2 | 2.8 | % | 1.9 | % |
| Attention problem (8 years) | Score | 0 | 79.0 | % | 81.2 | % |
|  |  | 1 | 21.0 | % | 18.8 | % |
| Adaptive problem (8 years) | Score | 0 | 66.5 | % | 69.7 | % |
|  |  | 1 | 26.5 | % | 23.7 | % |
|  |  | 2 | 6.0 | % | 5.8 | % |
|  |  | 3 | 1.0 | % | 0.8 | % |
| Conduct problem (8 years) | Score | 0 | 66.7 | % | 67.6 | % |
|  |  | 1 | 23.3 | % | 23.9 | % |
|  |  | 2 | 7.7 | % | 7.0 | % |
|  |  | 3 | 2.3 | % | 1.5 | % |
|  |  |  |  |  |  |  |
